# Pancreatic Involvement in Pediatric Inflammatory Bowel Disease

**DOI:** 10.3389/fped.2017.00218

**Published:** 2017-10-11

**Authors:** Javier Martín-de-Carpi, Melinda Moriczi, Gemma Pujol-Muncunill, Victor M. Navas-López

**Affiliations:** ^1^Unit for the Comprehensive Care of Paediatric Inflammatory Bowel Disease, Hospital Sant Joan de Déu, Barcelona, Spain; ^2^Pediatric Gastroenterology and Nutrition Unit, Hospital Materno Infantil, Málaga, Spain; ^3^IBIMA, Biomedical Institute of Málaga, Málaga, Spain

**Keywords:** inflammatory bowel disease, pancreatitis, pancreatic enzyme, extraintestinal manifestations, children

## Abstract

Inflammatory bowel disease (IBD) is a chronic condition that includes two clinical entities: Crohn’s disease and ulcerative colitis. Although both entities mainly affect the gastrointestinal tract are considered multisystemic diseases and may present extraintestinal manifestations involving other organs and systems. Pancreatic involvement in Pediatric IBD includes a heterogeneous group of clinical entities like acute pancreatitis, chronic pancreatitis, autoimmune pancreatitis, asymptomatic exocrine pancreatic insufficiency, increased pancreatic enzyme levels, structural abnormalities, and granulomatous inflammation. Although the mechanism for pancreatic involvement in IBD is not clearly elucidated, is important to keep in mind the association of these two entities in order to perform a prompt diagnosis and establish an appropriate treatment. The objective of this review is to update the available evidence on pancreatic involvement in children with IBD.

## Background

Inflammatory bowel disease (IBD) is a chronic and recurrent condition that encompasses two clinical entities: Crohn’s disease (CD) and ulcerative colitis (UC), which occur in a genetically susceptible individual. Numerous environmental factors influence the microbiota, giving rise to an excessive immune response, intestinal barrier function impairment and, ultimately, inflammation with subsequent tissue damage (1). Although both entities preferentially affect the gastrointestinal tract, the conditions can involve other organs and systems (skin, eyes, mouth, osteoarticular, hepatobiliary system, etc.), given that they are multisystem diseases (2).

We can find extraintestinal manifestations (EIM) that are the result of inflammatory or autoimmune phenomena and extraintestinal complications, secondary to metabolic abnormalities, adverse effects of drugs or anatomical abnormalities (3). The incidence of EIM in adult patients with IBD is around 30–40%. Two pediatric studies have shown similar results. The PediIBD Consortium Registry with a cohort of 1,649 patients showed that up to 29% of the patients had at least one EIM during the 15 years of follow-up (4), with 6% of them presenting before the diagnosis of IBD. Similar results were obtained from a series by Dotson et al. in the Pediatric IBD Collaborative Research Group Registry (5). This series included 1,009 patients, 28.2% of them experienced at least one EIM episode. It is noteworthy that those with more extensive or more severe disease had a greater risk of developing an EIM. Up to 130 EIMs were reported, but fortunately most of them were rare. The most common EIMs are musculoskeletal (axial and/or peripheral arthritis), mucocutaneous (erythema nodosum, pyoderma gangrenosum, or aphthous stomatitis), ocular (episcleritis and uveitis), and hepatobiliary. Pancreatic involvement in IBD may be due to EIM with a broad spectrum of presentations including acute pancreatitis (AP), chronic pancreatitis (CP), autoimmune pancreatitis (AIP), asymptomatic exocrine pancreatic insufficiency, increased pancreatic enzyme levels, structural abnormalities, and granulomatous inflammation (6). The pancreas can also be affected during the course of the disease due to drugs used for the treatment of IBD (drug-related pancreatitis or pancreatitis associated with total parenteral nutrition). The objective of this review is to update the available evidence on pancreatic involvement in children with IBD.

## Acute Pancreatitis

Acute pancreatitis is the most common pancreatic process associated with IBD. In fact, IBD is one of the five most important causes of AP in children (7). In adults, the incidence rate of AP is 1.2% for UC and 3.1% for CD and is 2.1 and 4.3 times more common in UC and CD, respectively, when compared with the general population (3). There are scarce published data on its actual incidence in children with IBD. In a retrospective study of 124 patients (97 cases of CD, 16 of UC, and 11 of IBD-U) by Le Large-Guiheneuf et al. (8), 18 patients (14.5%) presented symptomatic pancreatitis and 15 (12.5%) presented asymptomatic pancreatitis. The authors found an association between the inflammatory activity, the severity of the flare and the onset of AP. There was also an association with the drugs used (25%), the duodenal location of the CD (18%), and the presence of hepatobiliary complications (15%). In another study that included 101 children (79 cases of UC and 22 of CD), 4.5% of the patients with CD and 5.1% with UC developed AP (9). In a recent multicenter Italian study that recruited 649 patients (302 cases of UC, 297 of CD, and 50 of unclassified IBD), 27 patients (4.1%) showed increased levels of amylase and lipase, meeting only 11 (1.7%) the criteria for AP. The increased levels were more common in girls with colon involvement and active disease.

The most common situation is that AP appears once IBD has been diagnosed; however, in a small group of predominantly adult patients, AP was reported as the first clinical manifestation of IBD. Broide et al. (10) reported this condition in a multicenter study conducted in seven hospital centers in Israel. The study only included patients who had developed AP before the IBD diagnosis. The authors identified 30 episodes of pancreatitis in 12 patients (7 males, 10 children and 2 adults) from a cohort of 3,960 patients with IBD (3,500 adults and 460 children). The incidence rate in children was 2.17% at a mean age of 13 ± 4.8 years versus 0.06% for the adults. The mean time between the first AP episode and the IBD diagnosis was 24 (range 1–156) weeks. Of the 12 patients, 6 developed UC (4 cases of pancolitis), and 6 developed CD (2 cases of colonic, 2 of ileocolonic, and 2 of small intestine CD). Despite the fact that 9/12 (75%) patients had moderate-severe disease, the subsequent course was favorable, with a mean hospital stay of barely 3.5 days (range 0–18 days).

### Etiology

The causes of AP in patients with IBD can be summarized as follows: pharmacological causes, hepatobiliary diseases [cholelithiasis, duodenal obstruction and primary sclerosing cholangitis (PSC)], idiopathic causes, and granulomatous inflammation of the bile duct or ampulla of Vater (11).

#### Pharmacological Causes

Most of the drugs used for treating IBD could be responsible for the onset of AP (azathioprine, mercaptopurine, cyclosporine, sulfasalazine, 5-ASA, metronidazole, and steroids) mediated by different mechanisms (direct toxicity, hypersensitivity, dyslipidemia, and secondary hypercalcemia). Of the drugs included by Bai et al. (11), the American Gastroenterology Association has classified aminosalicylates and 6-MP (does not reference azathioprine) as drugs that are definitely associated with pancreatitis. The rest of the drugs were previously listed as probably associated (12). Toxic pancreatitis occurs in the first weeks of treatment. The symptoms are typical of AP, although they are usually mild and resolve after discontinuing the treatment (13). The risk of thiopurine-induced AP is low (14) and is more prevalent in children with CD (4.9%) than in those with UC (1.1%). The onset of AP with azathioprine does not represent an absolute contraindication for the use of mercaptopurine (13, 15). Before establishing the diagnosis of drug-induced pancreatitis, other causes for the AP must be ruled out. The definitive diagnosis of drug-induced pancreatitis requires three additional criteria to the International Study Group of Pediatric Pancreatitis: In Search for a Cure (INSPPIRE) criteria (16): a temporal sequence between the introduction of the drug and the onset of AP, the cessation of symptoms after discontinuing the treatment and a reappearance of AP after re-exposure. In practice, reintroduction of the drug is not standard practice (17).

#### Hepatobiliary Disease

The incidence of cholelithiasis in IBD is higher than in the general population, particularly in CD (there is no higher risk in UC than in the general population), although this complication is extremely rare in children. The risk of cholelithiasis is higher in patients with extensive ileal involvement and increases after ileal resection due to a reduction in enterohepatic circulation. The risk factors described for developing cholelithiasis in adults with CD include the following: previous intestinal resection (>30 cm), age (>50 years), involvement of the ileum and colon, duration of the disease (>10 years), number of hospitalizations (≥3), number of relapses (≥3), total parenteral nutrition, and length of hospital stay.

Duodenal involvement in CD is a rare cause of AP in adults (0.5–4% of all cases) and can be due to papillary obstruction, reflux of the duodenal content toward the pancreatic ducts or to the presence of duodenal-pancreatic fistulae or between duodenum and duct of Wirsung (1, 2, 14).

Most patients with PSC have UC (75–80%), and 3–10% of patients with IBD have associated PSC. Although patients with PSC can develop bile duct stricture, the onset of AP in these patients is extremely rare.

#### Idiopathic Causes

The association between colon involvement and AP development remains unexplained in many cases, and there are several hypotheses on this subject. A possible explanation could be the fact that the colon is the main source of bacteria responsible for pancreatic necrosis and that subtotal colectomy prior to the development of AP in rats decreased their mortality (10). In the study by Martinelli et al. (18), 3 of the 11 patients who presented AP did so at the onset of the disease. The systemic inflammatory nature and the hypercoagulability state of active IBD can predispose patients to developing AP, which would explain the correlation with the extent and severity of the flare-up (17). In addition, there is a risk in IBD of thromboembolic phenomena secondary to a hypercoagulability state due to an imbalance between procoagulant and anticoagulant factors and genetic determinants (17, 19). These thrombi can affect the gastrointestinal circulation, favoring pancreatic ischemia and the development of AP. Controlling the IBD through anti-TNF and the subsequent blocking of the TNF-mediated inflammatory cascade decreases the risk of AP (17, 20). The protein associated with pancreatitis (PAP-1), with anti-apoptotic and anti-inflammatory effects, inhibits NFkB activation, cytokine production and the expression of adhesion molecules in the inflamed tissue. An increase in the mRNA of PAP has been confirmed in active IBD, as well as high plasma PAP levels in patients when compared with the controls, both of which correlate with the clinical and endoscopic severity. Experimental data suggest that PAP-1 overexpression in the pancreas during trinitrobenzenesulfonic acid-induced colitis in murine models is the result of inflammatory stress that occurs in the pancreas of mice during experimental colitis. The failure of the intestinal mucosal barrier function plays a vital role in the course and development of AP, favoring bacterial translocation and development of infectious complications. Furthermore, changes in pancreatic epithelial tight-junctions are one of the earliest events that occur in AP in murine models. The *MYO9B* gene encodes for an unconventional myosin compound that can activate the domain of the GTPase protein (Rho-GAP), which regulates the tight-junction assembly and maintains the selectivity of the paracellular pathway in enterocytes (17, 21, 22). Genetic abnormalities in the MY09B gene have been found in both IBD and celiac disease, two entities where impairment of intestinal patency plays an important role. Polymorphisms of a single nucleotide in the MYO9B gene could explain the relationship between the two entities (IBD and AP) (17, 21, 22). It has also been postulated that abnormal MUC1 expression could be responsible for the pancreatic involvement in patients with IBD (3, 23). MUC1 is a transmembrane glycoprotein that is expressed on the apical membrane of the cells of the ductal epithelium of various organs and is also present in the colon epithelium of patients with IBD. MUC1 is abnormally expressed in innate immune cells. These MUC1-specific cells migrate not only to the colon but also to the pancreas of mice with IBD, which suggests that pancreatic inflammation could be the result of abnormal and proinflammatory MUC1 expression (23). Despite the fact that approximately 27–39% of patients with CD and 0–5% of those who have UC have high levels of anti-pancreas antibodies, the role of these antibodies in IBD-associated pancreatitis has not yet been elucidated and does not appear to be related to either the disease activity or with the onset of pancreatitis.

### Diagnosis

According to the Atlanta criteria and the INSPPIRE definitions (16), the diagnosis of AP is established with the presence of at least two of the following three criteria:

1. Clinical symptoms: abdominal pain, nausea, vomiting, and back pain.
2. Increase serum amylase/lipase levels at least three times the normal value.
3. Radiological abnormalities: pancreatic edema in ultrasonography or computed tomography.

In asymptomatic patients, increased pancreatic enzyme levels are not sufficient to establish the diagnosis of AP. Martinelli et al. (18) reported 16 cases (68.8% boys) of increased amylase/lipase levels without the patients meeting the AP criteria. This finding was correlated with the extent of the CD and the degree of inflammatory activity in the intestinal mucosa. The elevation of pancreatic enzymes (serum amylase/lipase levels expressed as the number of times the levels were above the normal value) was significantly lower in the IBD group without pancreatitis than in those of the AP group: amylase 1.6 (0.6–3.2) vs. 2.48 (1.5–4.1), *p* = 0.009; lipase 2.3 (0.6–8.4) vs. 13.6 (8.5–42.7), *p* = 0.001. A noteworthy fact is that, unlike what happened in those with AP, the results of the imaging tests were normal in the patients with increased pancreatic enzyme levels. The mechanism underlying this EIM is not well known but could be due to direct damage to the pancreas or perhaps to the increase in the passage of pancreatic enzymes from the intestinal lumen to the bloodstream due to an increase in intestinal permeability. The presence of macroamylasemia must be ruled out as the cause for increased plasma amylase levels (24). Regardless of the underlying mechanism for this impairment, pancreatic involvement must be screened, because 25% of patients who presented increased pancreatic enzyme levels at the onset developed AP in the following 6 months (3, 18). The prognosis was satisfactory in most cases.

### Treatment

The treatment for AP in patients with IBD does not differ from that of other causes of pancreatitis with the exception of the need for withdrawing the responsible drug (2, 25).

## Chronic Pancreatitis

Chronic pancreatitis is a chronic inflammatory process resulting from the destruction and fibrosis of exocrine pancreatic tissue and, in some cases, loss of endocrine pancreatic function. Unlike its acute form, which is defined by clinical criteria, CP is defined by morphological criteria. In adults, the incidence rate for IBD-associated CP varies (depending on the diagnostic criteria employed) between 1.2 and 1.5% (1:3 in women with UC and 1:1.3 in women with CD). IBD-associated CP is an extremely rare condition in children. Only two cases have been well documented, both after recurrent episodes of AP. One of the patients had the F1052V mutation in heterozygosity in the CFTR gene (18). In the other case, the results of the sweat test and the mutation study of the PRSS and SPINK1 genes (26) were normal. While AP can be considered an event, CP is a process and is occasionally the end result of repeated episodes of AP (27). The diagnosis of CP is based on a combination of clinical findings (abdominal pain, weight loss, and diabetes mellitus), functional impairment (documented exocrine pancreatic insufficiency), and imaging studies. Despite the fact that the etiological study was insufficient for ruling out the most common causes of recurrent pancreatitis, it is worth highlighting the importance of pancreatic function follow-up in these patients.

## Autoimmune Pancreatitis

Autoimmune pancreatitis is a rare entity and is most common in adults in whom two well-differentiated types are known. Type 1 AIP or lymphoplasmacytic sclerosing pancreatitis is the most common form, mostly affecting men in their 60s, and is currently considered the pancreatic manifestation of IgG4-related disease accompanied by other systemic manifestations such as sclerosing cholangitis, sclerosing sialadenitis and retroperitoneal fibrosis (3). Type 2 AIP or idiopathic duct centric pancreatitis is not characterized by increased IgG4 levels and affects adults in their 40s and 50s, has no predominance by sex and is the form frequently associated with IBD. The prevalence of IBD in patients with AIP is higher than in general population, mostly in UC patients.

In children, only 48 cases of AIP have been reported from a systematic review and cases collected from a multicenter group (INSPPITE) and those from Cliniques St-Luc (28–31). The mean age at diagnosis was 13 years (range 2–17). Both types of AIP can present as painless jaundice, weight loss, diabetes, and mild abdominal pain (3). Abdominal pain (43/47, 91%) and obstructive jaundice (20/47, 42%) were the most common symptoms at diagnosis. Diagnostic criteria for AIP include histology, imaging studies, serology (IgG4), involvement of other organs and response to treatment to steroids. IgG4 levels, one of the distinctive elements of AIP in adults, was high in only 9/40 (21%) cases. The results of the imaging tests were abnormal for all patients, with the following findings: overall or focal hypointense pancreatic hypertrophy (39/47, 83%), irregularity of the main pancreatic duct (29/43, 67%), and common bile duct narrowing (25/43, 58%). For those patients who underwent pancreatic biopsy, the most common findings consisted of a combination of lymphoplasmacytic inflammation, pancreatic fibrosis and ductal granulocytic infiltration (18/25%). The response to steroid treatment was fast. Twenty-seven percent of the patients developed other autoimmune phenomena: nine developed IBD (two CD and seven UC).

The AIP complications include diabetes (3/27, 11%) and exocrine pancreatic insufficiency with the need for replacement therapy with pancreatic enzymes (4/25, 16%). The findings recorded in this series suggest that AIP in children is a different subtype of pancreatitis than that described in adults and has the following characteristics:

1. High rate of abdominal pain at diagnosis.
2. Low rate of high IgG4 levels.
3. Ductal or parenchymal abnormalities in imaging tests.
4. Lymphoplasmacytic, granulocytic infiltrate with fibrosis.
5. Good response to steroid treatment.

## Silent Pancreatic Disorders

There have been reports of histological changes, pancreatic duct abnormalities and exocrine pancreatic insufficiency in patients with IBD but no pancreatic symptoms. These patients were likely underdiagnosed due to the symptoms (abdominal pain, diarrhea, etc.) being attributed to poor control of the inflammatory disease. Necropsy studies published in the 1950s showed that 38% of the 39 patients with CD showed pancreatic fibrosis and that 53% of the 86 patients with UC had chronic interstitial pancreatitis. Some 16.4% of the patients with UC and no previous history of alcohol consumption or previous episodes of AP showed pancreatic ducts disorders in the magnetic resonance cholangiopancreatography. Regardless of the imaging test findings or increased pancreatic enzyme levels, 4–18% of the patients with IBD presented exocrine pancreatic insufficiency, although two-thirds of the patients returned to normal fecal elastase levels at 4–6 months of progression, suggesting that the condition involved transient pancreatic insufficiency (1).

## Conclusion

Pancreatic involvement in IBD encompasses a heterogeneous group of clinical entities that can occur prior, together with or after the onset of IBD. The mechanisms responsible for pancreatic involvement in patients with IBD are not clearly elucidated.

Acute pancreatitis is the most common presentation but the increase in pancreatic enzyme levels is not sufficient to establish the diagnosis of AP in asymptomatic patients. It is important to follow diagnostic criteria for the different entities, as well as to establish the correct treatment in each case. We should reliably document drug-induced pancreatitis to avoid unnecessarily discontinuing the treatment, given the limited therapeutic options available.

## Author Contributions

All the authors have contributed to the review of published literature regarding the topic, writing the article, and the manuscript review.

## Conflict of Interest Statement

The authors declare that the research was conducted in the absence of any commercial or financial relationships that could be construed as a potential conflict of interest.
